# Identifying and Exploiting Potential miRNA-Disease Associations With Neighborhood Regularized Logistic Matrix Factorization

**DOI:** 10.3389/fgene.2018.00303

**Published:** 2018-08-07

**Authors:** Bin-Sheng He, Jia Qu, Qi Zhao

**Affiliations:** ^1^The First Affiliated Hospital, Changsha Medical University, Changsha, China; ^2^School of Information and Control Engineering, China University of Mining and Technology, Xuzhou, China; ^3^School of Mathematics, Liaoning University, Shenyang, China; ^4^Research Center for Computer Simulating and Information Processing of Bio-Macromolecules of Liaoning Province, Shenyang, China

**Keywords:** microRNA, disease, association prediction, neighborhood regularized, matrix factorization

## Abstract

With the rapid development of biological research, microRNAs (miRNA) have become an attractive topic because lots of experimental studies have revealed the significant associations between miRNAs and diseases. However, considering that experiments are expensive and time-consuming, computational methods for predicting associations between miRNAs and diseases have become increasingly crucial. In this study, we proposed a neighborhood regularized logistic matrix factorization method for miRNA-disease association prediction (NRLMFMDA) by integrating miRNA functional similarity, disease semantic similarity, Gaussian interaction profile kernel similarity, and experimentally validation of disease-miRNA association. We used Gaussian interaction profile kernel similarity to cover the shortage of the traditional similarity to make it more reasonable and complete. Furthermore, NRLMFMDA also considered the important influences of the neighborhood information and took full advantage of them to improve the accuracy of the miRNA-disease association prediction. We also improved the accuracy by giving higher weights to the known association data in the process of calculating the potential association probabilities. In the global and the local leave-one-out cross validation, NRLMFMDA got the AUCs of 0.9068 and 0.8239, respectively. Moreover, the average AUC of NRLMFMDA in 5-fold cross validation was 0.8976 ± 0.0034. All the three kinds of cross validations have shown significant advantages to a number of previous models. In the case studies of breast neoplasms, esophageal neoplasms and lymphoma according to known miRNA-disease associations in the recent version of HMDD database, there were 78, 80, and 74% of top 50 predicted related miRNAs verified to have associations with these three diseases, respectively. In the further case studies for new disease without any known related miRNAs and the previous version of HMDD database, there were also high proportions of the predicted miRNAs verified by experimental reports. All the validation experiment results have demonstrated the effectiveness and practicability of NRLFMDA to predict the potential miRNA-disease associations.

## Introduction

MicroRNAs (miRNAs) are a category of endogenous and short non-coding single-stranded RNAs (21~24 nucleotides) which could regulate the gene expression by targeting mRNAs for cleavage or translational repression at the posttranscriptional level (Ambros, 2001, 2004; Bartel, 2004; Meister and Tuschl, 2004). The first miRNA was found 20 years ago. And since then, people have discovered thousands of miRNAs in a wide variety of species (Jopling et al., 2005; Kozomara and Griffiths-Jones, 2011). Furthermore, more and more studies have found that the miRNAs play crucial roles at multiple stages of the biological processes (Lee et al., 1993; Chen et al., 2017b; Li et al., 2017), such as early cell growth, proliferation (Cheng et al., 2005), differentiation (Miska, 2005), development (Karp and Ambros, 2005), aging (Bartel, 2009), apoptosis (Xu et al., 2004), and so on. Additionally, the key regulatory roles of miRNAs have increasingly been paid attention to in the abnormal gene expression of biological cells. For example, the dysregulation of the miRNAs has been confirmed as a main reason of aberrant cell behavior by many studies (Griffiths-Jones et al., 2006). In the recent years, more and more experiments have been implemented to show that miRNAs have great connections with the various development processes of many human complex diseases (Lynam-Lennon et al., 2009; Meola et al., 2009; Huang et al., 2016b). For example, researches have implicated that miRNA-7a has clinical significance of high mobility group A2 in human gastric cancer. And Schulte et al. reported the capacity of miRNA-197 and miRNA-223 in predicting cardiovascular death and burden of future cardiovascular events in a large cohort of Coronary artery disease patients (Schulte et al., 2015). Besides, Thomas Thum et al. (Thum et al., 2008) showed that miR-21 affects the global cardiac structure and function through regulating the ERK–MAP kinase signaling pathway in cardiac fibroblasts. Therefore, identifying disease-related miRNAs is important and beneficial to the treatment, diagnosis, and prevention of a variety of clinically important disease. Nevertheless, identifying the associations between miRNAs and diseases with experimental methods is expensive and time-consuming. With the development of biological technology, lots of experiments have been implemented to produce vast numbers of miRNA-associated datasets. There is an urgent need for us to make further efforts to develop novel computational models for potential miRNAs-disease association prediction. In fact, many computational methods are well behaved in predicting miRNA-disease associations (Chen and Yan, 2013; Chen, 2015b; Chen et al., 2016a,g; Chen et al., 2018c). Therefore, further experimental studies can be more efficiently implemented by selecting the most promising associated miRNAs predicted by computational models.

Based on the assumption that functionally similar miRNAs are more likely to have associations with phenotypically similar diseases, many computational approaches have been introduced for the identification of miRNA-disease associations (Bandyopadhyay et al., 2010; Jiang et al., 2010; Liu et al., 2016b; Pasquier and Gardès, 2016; Zeng et al., 2016b; Zou et al., 2016; Chen and Huang, 2017; Chen et al., 2017a,c,d; You et al., 2017; Chen et al., 2018a,b,d,e,f; Tang et al., 2018). A hypergeometric distribution-based model was proposed by Jiang et al. (2010). Through using the human known disease-miRNA association network, disease phenotype similarity network and miRNA functional similarity network, this model gave the prediction of miRNA-disease associations. But there was a high proportion of false positive and false negative samples in the miRNA-target associations set on which this method extremely depended. Shi et al. (2013) proposed a random walk algorithm-based model in protein-protein interaction (PPI) network under the assumption that miRNAs have closer associations with the diseases that are more correlated to the miRNA targets. They obtained potential miRNA-disease associations by the comprehensive consideration of miRNA–target interactions, disease–gene associations and PPIs. Mørk et al. (2014) presented a miRPD method by integration of miRNA-protein association scores, protein-disease association scores and the shared proteins between miRNAs and diseases to obtain the best scoring protein connections between miRNA-disease pairs. Xu et al. (2014) introduced a miRNA prioritization model by the integrationof known disease–gene associations and miRNA-target interactions. It is worthy mentioning that the model is independent of the experimentally verified miRNA-disease associations. Instead, they need to calculate the similarity between miRNA targets and disease genes. Nonetheless, the aforementioned methods could not provide sufficiently accurate prediction results due to the incomplete disease-gene association network or/and the miRNA-target interactions with high false positive and false negative samples.

Xuan et al. (2013a) constructed a computational method called HDMP for the identification of miRNA-disease associations based on the experimentally verified miRNA-disease associations, miRNA functional similarity, disease semantic similarity and disease phenotype similarity. According to miRNAs with similar functions are normally related to similar diseases and vice versa, they used the *k* nearest neighbors of miRNAs for estimating more reliable relevance scores of the unlabeled miRNAs. To overcome the shortages of the previous methods, it assigned higher weights to members in the same miRNA cluster when they calculated the miRNA functional similarity. However, the HDMP cannot prioritize miRNAs(diseases) for diseases(miRNAs) that have no known related miRNAs(diseases). Additionally, the performance of HDMP could not better than most of previous models which were calculated based on the global network similarity measure. A global network similarity-based computational model was proposed by Chen et al. (2012b) called RWRMDA, which used the random walk method based on the dataset of human known miRNA–disease associations and miRNA functional similarity. We can see that RWRMDA has excellent prediction performance through cross-validation and case studies of several important human complex cancers. However, there is a non-negligible limitation that it could not work for diseases without any known associated miRNAs. Chen et al. (2016f) developed another computational approach of WBSMDA by integrating the Gaussian interaction profile kernel similarity, miRNA functional similarity, disease semantic similarity, and miRNA-disease associations for the prediction of potential miRNAs-diseases associations. WBSMDA could effectively predict disease(miRNA)-related miRNAs(diseases) that without known related miRNAs(diseases). Recently, Chen et al. (2016d) developed a novel computational model named HGIMDA, which had superior performance compared with four classical methods (WBSMDA, RLSMDA, RWRMDA, and HDMP).

Nowadays, machine learning has been applied in extensive scientific fields, and it is highly effective for most of the research problems (Chen et al., 2012a, 2015c, 2016c; Wong et al., 2015; Huang et al., 2016b). Therefore, more and more studies have focused on it. For instance, Xu et al. (2011) proposed a computational model, named miRNA-target dysregulated network (MTDN), which combined miRNA-target interactions and expression pattern of miRNAs and mRNAs. In the model, the support vector machine (SVM) classifier was constructed to distinguish positive miRNA-disease associations from negative ones by extracting the feature of network topologic information. It is known that negative miRNA-disease associations are difficult to obtain, and the ambiguity caused by negative samples usually affects the accuracy of the supervised. Chen et al. (Chen and Yan, 2014) provided RLSMDA, a computational model in which they used semi-supervised learning to predict potential disease-related miRNAs by the consideration of disease semantic similarity, miRNA functional similarity, and known miRNA-disease associations. Furthermore, RLSMDA could also predict disease(miRNA)-related miRNAs(diseases) without any known miRNAs(diseases) and avoid the problem of using negative miRNA-disease associations. However, the ways of combining the classifiers in different spaces together and the selection of parameters for RLSMDA would greatly influence the prediction result. Based on known miRNA-disease associations, Chen et al. (2015b) further developed a computational model of RBMMMDA by presenting restricted Boltzmann machine (RBM). RBMMMDA is a two-layer (visible and hidden) undirected graphical model, which can not only obtain new miRNA-disease associations, but also corresponding association types. Nevertheless, it is difficult to make decision on the parameter values.

In our proposed method, we introduced a novel matrix factorization computational approach, namely neighborhood regularized logistic matrix factorization for miRNA-disease association prediction (NRLMFMDA). In consideration of the effectiveness of the classical method with integrated similarities, we combined the Gaussian interaction profile kernel similarity and the modified matrix factorization to get a more accuracy prediction result. Based on the known miRNA-disease associations, disease semantic similarity, miRNA functional similarity, and Gaussian interaction profile kernel similarity, the proposed method focuses on predicting the probability that a miRNA would be associated with a disease by mapping a miRNA and a disease to a shared low dimensional latent space as two latent vectors. Additionally, we also studied the local structure of the association data to further improve the prediction accuracy by exploiting the influences of the neighbors which were from the most similar miRNAs and most similar diseases. Moreover, the proposed approach assigned higher importance level to the nearest neighbors for avoiding noisy information. Furthermore, we used global LOOCV, local LOOCV, and 5-fold cross validation to evaluate the effectiveness of NRLMFMDA. As a result, the AUCs of global and local LOOCV are 0.9068 and 0.8239, respectively. By adopting 5-fold cross validation, NRLMFMDA model obtained the average AUC of 0.8976 ± 0.0034. In three types of case studies, we tested the prediction effect of NRLMFMDA for known diseases in the recent version of HMDD database, new diseases without any known related miRNAs and known disease based on previous version of HMDD database, respectively. As a result, most of the predicted miRNAs have been confirmed by recent experimental reports. Thus, we can conclude that NRLMFMDA is a useful tool in predicting potential miRNA-disease associations.

## Materials and methods

### Human miRNA-disease association

For convenience, we have built an adjacency matrix *Y* ∈ *R*^*m*×*n*^ to formalize the known miRNA-disease associations that acquired from the HMDD v2.0 database (Li et al., 2014). The known miRNA-disease associations dataset used in this paper includes 5430 distinct experimentally confirmed miRNA-disease between 383 diseases and 495 miRNAs, *m* and *n* were expressed as the miRNAs and diseases numbers in the dataset. Then we stored the known miRNA-disease association information into the matrix *Y*. If a miRNA *r*_*i*_ has been experimentally verified to be associated with a disease*d*_*j*_, then *y*_*ij*_ equals to 1, otherwise 0.

### miRNA functional similarity

The miRNA functional similarity was calculated according to the method proposed by Wang et al. (2010) by the consideration of miRNAs with functional similar tend to be interacted with semantic similar diseases, and vice versa (Goh et al., 2007; Lu et al., 2008). Owing to their excellent work, we can download the miRNA functional similarity data from http://www.cuilab.cn/files/images/cuilab/misim.zip. The matrix *MS* was constructed to represent the miRNA functional similarity. The element *MS*(*r*_*i*_, *r*_*j*_) represented the value of similarity between the miRNA *r*_*i*_ and the miRNA*r*_*j*_.

### Disease semantic similarity model 1

We constructed a Directed Acyclic Graph (DAG) to describe the diseases according to the MeSH descriptors downloaded from the National Library of Medicine (http://www.nlm.nih.gov/) (Chen, 2015a; Chen et al., 2015a, 2016a,e; Huang et al., 2016a). Then we defined the contribution of disease *d* in DAG(*D*) to the semantic value of disease *D* as follows:

(1) $$\begin{array}{l} \{\begin{array}{c} D1_{D}\left(d\right)=1\;if\;d=D\\ D1_{D}\left(d\right)=\max\left\{\Delta^{*}D1_{D}\left(d^{\prime }\right)|d^{\prime }\in children\;of\;d\right\}if\;d\neq D \end{array} \end{array}$$

where Δ is the semantic contribution decay factor and we set the value of Δ to 0.5 (Xuan et al., 2013b). The self-semantic value of disease *D* is defined as follows:

(2) $$\begin{array}{l} DV1\left(D\right)=\sum_{d\in T\left(D\right)}D1_{D}\left(d\right) \end{array}$$

where *T*(*D*) represents *D* itself and all its ancestral nodes. According to the observation that two diseases with larger shared part of their DAGs have larger similarity score, the semantic similarity score between disease *d*_*i*_ and *d*_*j*_ are defined as follows:

(3) $$\begin{array}{l} SS1\left(d_{i},d_{j}\right)=\frac{\sum_{t\in T\left(d_{i}\right)⋂T\left(d_{j}\right)}\left(D1_{d_{i}}\left(t\right)+D1_{d_{j}}\left(t\right)\right)}{DV1\left(d_{i}\right)+DV1\left(d_{j}\right)} \end{array}$$

### Disease semantic similarity model 2

Different from disease semantic similarity model 1, we considered that assigning the same contribution value to the diseases in the same layer of DAG(*D*) was not reasonable. Actually, a more specific disease which appears in less DAGs contributes to the semantic similarity of disease *D* at a higher contribution level. So we made definition for the contribution of disease *d* in DAG(*D*) to the semantic value of disease *D* as follows:

(4) $$\begin{array}{l} D2_{D}\left(d\right)=-\log[\text{the number of DAGs including t}/\\ \text{ the number of diseases}] \end{array}$$

We gave definition of the semantic similarity between disease *d*_*i*_ and *d*_*j*_ are the proportion of the summing contributions of their shared ancestor nodes and themselves to them in all the contributions of their ancestor nodes and themselves defined as the disease semantic similarity model 1.

(5) $$\begin{array}{l} SS2\left(d_{i},d_{j}\right)=\frac{\sum_{t\in T\left(d_{i}\right)⋂T\left(d_{j}\right)}\left(D2_{d_{i}}\left(t\right)+D2_{d_{j}}\left(t\right)\right)}{DV2\left(d_{i}\right)+DV2\left(d_{j}\right)} \end{array}$$

### Gaussian interaction profile kernel similarity

Considering that Gaussian kernel function is one of the Radial Basis function whose value depends only on the distance from the origin, we constructed Gaussian interaction profile kernel similarity as another similarity algorithm that different from disease semantic similarity and miRNA functional similarity (Van et al., 2011; Chen et al., 2016b). Our definition of vector *IV*(*d*_*i*_) and *IV*(*r*_*j*_) are the *i*^*th*^ row and *j*^*th*^ column of adjacent matrix *Y* which represents whether the disease or the miRNA associated with each of the miRNAs or the diseases. Accordingly, the Gaussian interaction profile kernel similarity of diseases and miRNAs can be computed as follows:

(6) $$\begin{array}{lll} GD\left(d_{i},d_{j}\right) & = & \exp\left(-\beta_{d}{\left||IV\left(d_{i}\right)-IV\left(d_{j}\right)|\right|}^{2}\right) \end{array}$$

(7) $$\begin{array}{lll} GR\left(r_{i},r_{j}\right) & = & \exp\left(-\beta_{r}{\left||IV\left(r_{i}\right)-IV\left(r_{j}\right)|\right|}^{2}\right) \end{array}$$

where adjustment coefficient β_*d*_ and β_*r*_ for the kernel bandwidth can be denoted as follows:

(8) $$\beta_{d}={\beta^{\prime }}_{d}/\left(\frac{1}{n}\sum_{i=1}^{n}{\left\|IV(d_{i})\right\|}^{2}\right)$$

(9) $$\beta_{r}={\beta^{\prime }}_{r}/\left(\frac{1}{m}\sum_{i=1}^{m}{\left\|IV(r_{i})\right\|}^{2}\right)$$

where ${\beta \prime }_{d}$ and ${\beta \prime }_{r}$ are the original bandwidths and both of them were set 1 according to the previous literature (Chen and Yan, 2013).

### Integrated similarity for MiRNAs and diseases

As mentioned above, a Directed Acyclic Graph (DAG) was introduced to describe a disease based on the MeSH descriptors. Disease semantic similarity was calculated according to the assumption that the two diseases with larger shared area of their DAGs may have greater similarity score. In fact, for the specific disease that without DAG, we cannot calculate the semantic similarity between the specific disease and other diseases. Thus, for disease pairs that have no semantic similarity, we used Gaussian interaction profile kernel similarity score to define their similarity. We gave a definition of integrated disease similarity by the combination of disease semantic similarity and Gaussian interaction profile kernel similarity for disease. Specifically, if disease *d*_*i*_ and *d*_*j*_ have semantic similarity, the integrated disease similarity can be defined as the average of *SS1* and *SS2*, otherwise we would attach the value of Gaussian interaction profile kernel similarity for disease to the integrated disease similarity. The formulations show as follows:

(10) $$\begin{array}{l} SD\left(d_{i},d_{j}\right)=\{\begin{array}{cc} \frac{SS1\left(d_{i},d_{j}\right)+SS2\left(d_{i},d_{j}\right)}{2} & d_{i}\text{ and }d_{j}\text{ has semantic similarity}\\ GD\left(d_{i},d_{j}\right) & \text{otherwise} \end{array} \end{array}$$

In the same way, we made a definition for integrated miRNA similarity through combining miRNA functional similarity and Gaussian interaction profile kernel similarity for miRNA. we obtained the integrated miRNA similarity as follows:

(11) $$\begin{array}{l} SR\left(r_{i},r_{j}\right)=\{\begin{array}{cc} MS\left(r_{i},r_{j}\right) & r_{i}\text{ and }r_{j}\text{ has functional similarity}\\ GR\left(r_{i},r_{j}\right) & \text{otherwise} \end{array} \end{array}$$

### NRLMFMDA

**Figure 1:**
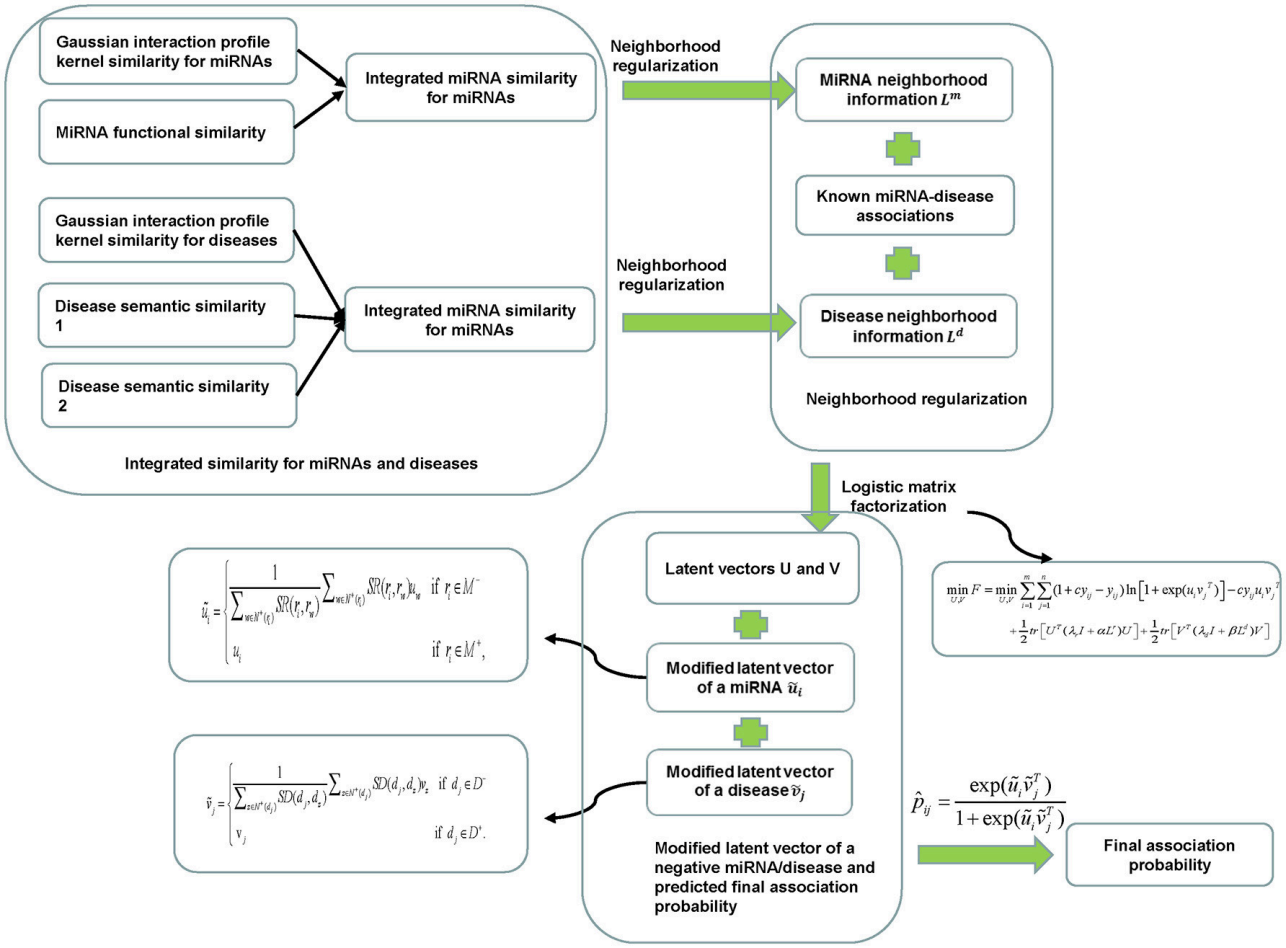
Flowchart of NRLMFMDA model to predict the potential miRNA-disease associations based on the known associations in HMDD database.

In this study, we proposed a neighborhood regularized logistic matrix factorization method for miRNA-disease association prediction (NRLMFMDA) by integrating known miRNA-disease associations, miRNA functional similarity, disease semantic similarity, and Gaussian interaction profile kernel similarity (see Figure 1). As far as we have known, the matrix factorization has been applied to recommender systems and obtained successful association prediction results currently. For example, logistic matrix factorization (LMF) (Johnson, 2014) has been demonstrated to be effective for personalized recommendations. Therefore, the probability of the association between a miRNA and a disease can be computed based on it. In details, we mapped the diseases and the miRNAs into a shared latent space with a dimensionality *r* which is far lower than the minimum of *m* and *n*. The latent space vectors $u_{i}\in R^{1\times r}$ and $v_{j}\in R^{1\times r}$ are used to represent the properties of the miRNA *r*_*i*_ and the disease*d*_*j*_, respectively. For simplicity, we further denote the latent vectors of all miRNAs and all diseases by *U* ∈ *R*^*m*×*r*^ and *V* ∈ *R*^*n*×*r*^ respectively, where *u*_*i*_ is the *i*^th^ row in *U* and *v*_*j*_ is the *j*^th^ row in *V*. Simultaneously, the probability distributions of *U* and *V* are assumed as Gaussian distributions with zero-means and their variances are set as $\sigma_{r}^{2}$ and $\sigma_{d}^{2}$, respectively. Their formulations are shown as follows:

(12) $$\begin{array}{l} p\left(U|\sigma_{r}^{2}\right)=\overset{m}{\underset{i=1}{\prod }}N\left(u_{i}|0,\sigma_{r}^{2}I\right),p\left(V|\sigma_{d}^{2}\right)=\overset{n}{\underset{j=1}{\prod }}N\left(v_{j}|0,\sigma_{d}^{2}I\right) \end{array}$$

where *I* denotes the identity matrix. Afterwards, based on the Bayesian theorem, we know that

(13) $$\begin{array}{l} p\left(U,V|Y,\sigma_{r}^{2},\sigma_{d}^{2}\right)\propto p\left(Y|U,V\right)p\left(U|\sigma_{r}^{2}\right)p\left(V|\sigma_{d}^{2}\right). \end{array}$$

Based on the assumption that all the training examples are independent, we denoted the probability of associations under the condition of *U* and *V* as follows:

(14) $$\begin{array}{l} p\left(Y|U,V\right)=\overset{m}{\underset{i=1}{\prod }}\overset{n}{\underset{j=1}{\prod }}p_{ij}^{cy_{ij}}{\left(1-p_{ij}\right)}^{\left(1-y_{ij}\right)} \end{array}$$

where we denote the probability *p*_*ij*_ of the association between miRNA *r*_*i*_ and disease *d*_*j*_as follows:

(15) $$\begin{array}{l} p_{ij}=\frac{\exp\left(u_{i}v_{j}^{T}\right)}{1+\exp\left(u_{i}v_{j}^{T}\right)} \end{array}$$

And the known associations between diseases and miRNAs are assigned with higher importance levels of *c* (*c* > 1) which is empirically set to 5 in experiment so that we could get more accurate predictions with the help of the trustworthy data. Then, we made the log form on the both side of the formula (13) as follows:

(16) $$\begin{array}{lll} \log p\left(U,V|Y,\sigma_{r}^{2},\sigma_{d}^{2}\right) & = & \overset{m}{\underset{i=1}{\sum }}\overset{n}{\underset{j=1}{\sum }}cy_{ij}u_{i}{v_{j}}^{T}\\ -\left(1+cy_{ij}-y_{ij}\right)\log\left[1+\exp\left(u_{i}v_{j}^{T}\right)\right]\\ -\frac{1}{2\sigma_{r}^{2}}\overset{m}{\underset{i=1}{\sum }}||u_{i}|{|}_{2}^{2}-\frac{1}{2\sigma_{d}^{2}}\overset{n}{\underset{j=1}{\sum }}||v_{j}|{|}_{2}^{2}+C \end{array}$$

where *C* is a constant. We maximized the posterior distribution to obtain the most possible *U* and *V*. And it is equivalent to the problem as follows:

(17) $$\begin{array}{l} \underset{U,V}{\min}\overset{m}{\underset{i=1}{\sum }}\overset{n}{\underset{j=1}{\sum }}\left(1+cy_{ij}-y_{ij}\right)\log\left[1+\exp\left(u_{i}v_{j}^{T}\right)\right]\\ -cy_{ij}u_{i}{v_{j}}^{T}+\frac{\lambda_{r}}{2}||U|{|}_{F}^{2}+\frac{\lambda_{d}}{2}||V|{|}_{F}^{2} \end{array}$$

where$\lambda_{r}=\frac{1}{\sigma_{r}^{2}},\lambda_{d}=\frac{1}{\sigma_{d}^{2}}$, and ||•||_*F*_ is the Frobenius norm of a matrix. We solved this searching minimum problem with an alternating gradient descent method (Johnson, 2014). Because the neighborhoods of a miRNA or a disease have strong associations, the nearest miRNAs and diseases can provide the most useful information about how to find the reasonable way to factorize the logical matrix. Therefore, our object is to minimize the distances between *d*_*i*_ and its nearest neighbors in set *N*(*d*_*i*_) which is formed by *K*_1_ nearest neighbors of the disease*d*_*i*_. The same to miRNA*r*_*j*_, *N*(*r*_*j*_)is the set formed by *K*_1_ nearest neighbors of the miRNA*r*_*j*_. *K*_1_is empirically set to 5 in experiment. We used the adjacency matrix *A* and *B* to represent the neighborhood information, and their elements *a*_*iu*_ and *b*_*jv*_ are defined as follows:

(18) $$\begin{array}{lll} a_{i\mu } & = & \{\begin{array}{cc} SR\left(r_{i},r_{\mu }\right) & if\;r_{\mu }\in N\left(r_{i}\right)\\ 0 & \text{otherwise} \end{array} \end{array}$$

(19) $$\begin{array}{lll} b_{jv} & = & \{\begin{array}{cc} SD\left(d_{j},d_{v}\right) & if\;d_{v}\in N\left(d_{j}\right)\\ 0 & \text{otherwise} \end{array} \end{array}$$

Based on them, we aimed to minimize the following functions:

(20) $$\begin{array}{l} \;\frac{\alpha }{2}\overset{m}{\underset{i=1}{\sum }}\overset{m}{\underset{\mu =1}{\sum }}a_{i\mu }||u_{i}-u_{\mu }|{|}_{F}^{2}\\ =\frac{\alpha }{2}\left[\overset{m}{\underset{i=1}{\sum }}\left(\overset{m}{\underset{\mu =1}{\sum }}a_{i\mu }\right)u_{i}u_{i}^{T}+\overset{m}{\underset{\mu =1}{\sum }}\left(\overset{m}{\underset{i=1}{\sum }}a_{i\mu }\right)u_{\mu }u_{\mu }^{T}\right]\\ \;-\frac{\alpha }{2}tr\left(U^{T}AU\right)-\frac{\alpha }{2}tr\left(U^{T}A^{T}U\right)\\ =\frac{\alpha }{2}tr\left(U^{T}L^{r}U\right) \end{array}$$

(21) $$\begin{array}{l} \frac{\beta }{2}\overset{n}{\underset{j=1}{\sum }}\overset{n}{\underset{v=1}{\sum }}b_{jv}||v_{j}-v_{v}|{|}_{F}^{2}=\frac{\beta }{2}tr\left(V^{T}L^{d}V\right) \end{array}$$

where $L^{r}=\left(D^{r}+{\tilde{D}}^{r}\right)-\left(A+A^{T}\right)$ and $L^{d}=\left(D^{d}+{\tilde{D}}^{d}\right)-\left(B+B^{T}\right)$. In the two formulations, *D*^*r*^, ${\tilde{D}}^{r}$, *D*^*d*^,and ${\tilde{D}}^{d}$ are diagonal matrices and their diagonal elements are ${}_{ii}^{r}=\sum_{\mu =1}^{m}a_{i\mu }$, ${\tilde{D}}_{\mu \mu }^{r}=\sum_{i=1}^{m}a_{i\mu }$, $D_{jj}^{d}=\sum_{v=1}^{n}b_{jv}$, and ${\tilde{D}}_{jj}^{d}=\sum_{j=1}^{n}b_{jv}$, respectively. According to the analysis above, the integrated formulation to minimize the objective function *F* is as follows:

(22) $$\begin{array}{lll} \underset{U,V}{\min}F & = & \underset{U,V}{\min}\overset{m}{\underset{i=1}{\sum }}\overset{n}{\underset{j=1}{\sum }}\left(1+cy_{ij}-y_{ij}\right)\ln\;\left[1+\exp\left(u_{i}{v_{j}}^{T}\right)\right]\\ -cy_{ij}u_{i}{v_{j}}^{T}+\frac{1}{2}tr\left[U^{T}\left(\lambda_{r}I+\alpha L^{r}\right)U\right]\\ +\frac{1}{2}tr\left[V^{T}\left(\lambda_{d}I+\beta L^{d}\right)V\right] \end{array}$$

However, the alternating gradient descent method needs the partial differential of *F* with respect to *U* and *V*, so they are computed and simplified as follows:

(23) $$\begin{array}{lll} \frac{\partial F}{\partial U} & = & PV+\left(c-1\right)\left(Y*P\right)V-cYV+\left(\lambda_{r}I+\alpha L^{r}\right)U\\ \frac{\partial F}{\partial V} & = & P^{T}U+\left(c-1\right)\left(Y^{T}*P^{T}\right)U-cY^{T}U+\left(\lambda_{d}I+\beta L^{d}\right)V \end{array}$$

where *P* ∈ *R*^*m*×*n*^ is the matrix with elements *p*_*ij*_ in equation (10) and ∗ represents the Hadamard product. The gradient step size is chosen based on the AdaGrad algorithm (Duchi et al., 2011). In the experiments, we selected the dimensionality of the latent space *r* from {50, 100}. Simultaneously, we set λ_*r*_ = λ_*d*_ and chose the values from {2^−5^, 2^−4^, …, 2^1^}. Neighborhood regularization parameters α and β were selected from {2^−5^, 2^−4^, …, 2^2^} and {2^−5^, 2^−4^, …, 2^0^}. The optimal learning rate γ was selected from {2^−3^, 2^−2^, …, 2^0^}.

In the training procedure, the new diseases and new miRNAs are learned based on the mixed negative samples (including potential positive miRNA-disease associations) which will lead to a bias on the prediction results. Therefore, before obtaining the final probabilities with the learned *U* and *V* above, we further improved the prediction accuracy for new diseases or new miRNAs by replacing the latent vectors of negative samples with the linear combination of its nearest positive neighbors. For a miRNA *r*_*i*_ in negative set *M*^−^ which is the set of new miRNAs without any known related diseases, we denoted its *K*_2_ nearest neighbors in positive set *M*^+^ by $N^{+}\left(r_{i}\right)$. And for a disease *d*_*j*_ in negative set *D*^−^ which is the set of new diseases without any known related miRNAs, we denoted its *K*_2_ nearest neighbors in positive set *D*^+^ by $N^{+}\left(d_{j}\right)$, where *K*_2_ is empirically set to 5 in experiment. Hence, the modified association probability is represented as follows:

(24) $$\begin{array}{l} {\hat{p}}_{ij}=\frac{\exp\left({ũ}_{i}{ṽ}_{j}^{T}\right)}{1+\exp\left({ũ}_{i}{ṽ}_{j}^{T}\right)} \end{array}$$

Where,

(25) $$\begin{array}{lll} {ũ}_{i} & = & \{\begin{array}{cc} \frac{1}{\sum_{w\in N^{+}\left(r_{i}\right)}SR\left(r_{i},r_{w}\right)}\sum_{w\in N^{+}\left(r_{i}\right)}SR\left(r_{i},r_{w}\right)u_{w} & if\;r_{i}\in M^{-}\\ u_{i} & if\;r_{i}\in M^{+}, \end{array}\\ {ṽ}_{j} & = & \{\begin{array}{cc} \frac{1}{\sum_{z\in N^{+}\left(d_{j}\right)}SD\left(d_{j},d_{z}\right)}\sum_{z\in N^{+}\left(d_{j}\right)}SD\left(d_{j},d_{z}\right)v_{z} & if\;d_{j}\in D^{-}\\ v_{j} & if\;d_{j}\in D^{+}. \end{array} \end{array}$$

The modified latent vectors are helpful to overcome the bias due to using the uncertain negative samples to train the latent vectors of miRNAs and diseases in negative sets.

## Results

### Performance evaluation

**Figure 2:**
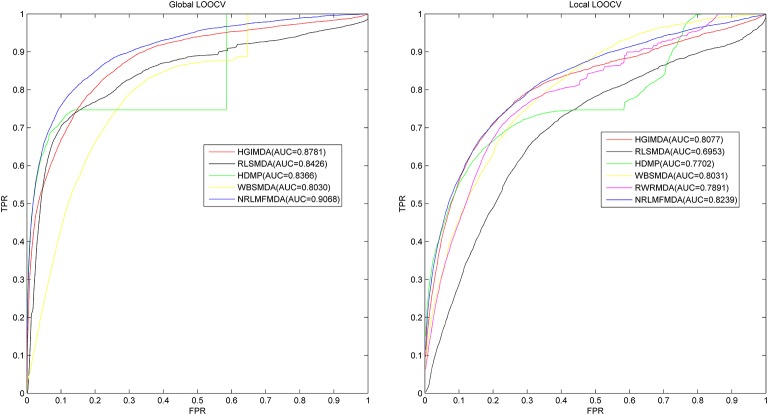
AUC of global LOOCV (left) compared with HGIMDA, RLSMDA, HDMP, and WBSMDA; AUC of local LOOCV (right) compared with HGIMDA, RLSMDA, HDMP, WBSMDA, and RWRMDA. As a result, NRLMFMDA achieved AUCs of 0.9068 and 0.8239 in the global and local LOOCV, which exceed all the previous classical models.

Leave-one-out cross validation (LOOCV) and 5-fold cross validation were applied to evaluate the performance of NRLMFMDA. And the LOOCV was implemented in two ways. (1) Based on the experimentally confirmed miRNA-disease associations in HMDD v2.0 database, Global LOOCV was used to evaluate the performance of NRLMFMDA. The “global” means that each one of the known miRNA-disease associations will be left out in turn to be considered as candidate association which are the unconfirmed miRNA-disease associations. Then after calculating prediction association scores of all the miRNA-disease pairs by NRLMFMDA, we compared the score of each test sample with all the candidate ones to observe whether its rank was above the threshold which was given in advance. (2) Unlike the Global LOOCV, Local LOOCV only compared the score of each test sample with the candidate samples composed of all the miRNA-disease pairs whose miRNAs did not have any known associations with the investigated disease. And if the rank of the test association exceeded the threshold which was given ahead of time, the model was considered to successfully predict this miRNA-disease association. Further, we drew Receiver operating characteristics (ROC) curve by plotting the true positive rate (TPR, sensitivity) vs. the false positive rate (FPR, 1-specificity) at different thresholds. Sensitivity refers to the percentage of the positive samples correctly identified among all the positives. Meanwhile, specificity denotes the percentage of negative samples correctly identified among all the negatives. After that, the prediction ability of NRLMFMDA would be evaluated by Area under the ROC curve (AUC). AUC = 1 indicates the prediction performance of NRLMFMDA is perfect; AUC = 0.5 indicates the prediction performance of NRLMFMDA is random. The results showed that NRLMFMDA obtained the AUC of 0.9068 and 0.8239 in global and local LOOCV, respectively (see Figure 2). The AUC results implied that the NRLMFMDA had shown reliable and effective prediction performance for potential miRNA–disease association prediction. However, HGIMD, RLSMDA, HDMP, and WBSMDA obtained the AUC of 0.8781, 0.8426, 0.8366 and 0.8030 in global LOOCV, respectively. In local LOOCV, their AUCs are 0.8077, 0.6953, 0.7702, and 0.8031, respectively. Differently, RWRMDA only has AUC of local LOOCV (0.7891) which is one of its defects because it cannot uncover the missing associations for all the diseases simultaneously. Therefore, in comparison with the previous methods, we can intuitively observe the improvement of predicting the miRNA-disease associations with NRLMFMDA.

Additionally, we also implemented 5-fold cross validation to evaluate the prediction effectiveness of NRLMFMDA. We firstly divided the known miRNA-disease associations into five parts randomly. Then, one of the five parts was treated as test samples and the remaining four parts were regarded as training samples in turn. In the same way as LOOCV, the miRNA-disease pairs without known evidence of association were regarded as candidate samples. Afterwards, the scores of test samples were taken out to compare with the scores of candidate samples, and we finally acquired their rankings. This procedure was repeated 100 times randomly to make validation more accuracy. In comparison with RLSMDA, HDMP, and WBSMDA whose average AUCs were 0.8569 ± 0.0020, 0.8342 ± 0.0010 and 0.8185 ± 0.0009 respectively, the average AUC of NRLMFMDA in 5-fold cross validation was 0.8976 ± 0.0034 which further confirmed the effectiveness and superiority for predicting potential miRNA-disease associations. At last, in order to obtain a clear knowledge of the predictability performance of NRLMFMDA. We listed evaluation result of NRLMFMDA and other several typical models in global LOOCV, local LOOCV as well as 5-fold cross validation by using tabular format (see Table 1).

**Table 1:** Performance evaluation comparison between NRLMFMDA and other several typical models in global LOOCV, local LOOCV and 5-fold cross validation based on known miRNA-disease associations.

**Model**	**The AUC in global LOOCV**	**The AUC in local LOOCV**	**The AUC in 5-fold cross validation**
NRLMFMDA	0.9068	0.8239	0.8976 ± 0.0034
HGIMDA	0.8781	0.8077	N/A
RLSMDA	0.8426	0.6953	0.8569 ± 0.0020
HDMP	0.8366	0.7702	0.8342 ± 0.0010
WBSMDA	0.8030	0.8031	0.8185 ± 0.0009
RWRMDA	N/A	0.7891	N/A

### Case studies

Based on another two miRNA-disease association databases, namely dbDEMC (Yang et al., 2010) and miR2Disease (Jiang et al., 2009), we studied three common major diseases of human beings to verify the prediction results of NRLMFMDA. The dataset of 5430 known miRNA-disease associations from HMDD v2.0 was treated as training set. For each disease, all candidate miRNAs would be ranked in the light of their predicted scores and the top 50 predicted miRNAs would be confirmed using another two miRNA-disesase association databases (i.e., dbDEMC and miR2Disease). It is worth noting that only candidate miRNAs that without known associations with investigated disease were ranked and confirmed. Therefore, there is no overlap between the training samples and the prediction lists and none of the top 50 predicted miRNAs existed in HMDD v2.0. We ulteriorly observed the number of the verified miRNAs in the top 10, top 20 and top 50 ones which are related with the three diseases respectively in the two databases.

Breast cancer is the worldwide women's health threatening, and it has caused large quantity of death in female all over the world. More than 80% of breast cancers are hormone-receptor positive in the western world (Van et al., 2014). About 232,340 new cases of invasive breast cancer including 39,620 breast cancer deaths occurred among women of America in 2013. At present, more and more researchers have paid attention to the original etiology of miRNAs in breast cancers and increasing number of evidences show that several miRNAs are closely related to breast cancer and play important roles in the tumorigenesis of breast cancer. For example, among the differentially expressed miRNAs, miR-10b, miR-125b, miR145, miR-21, and miR-155 showed as the most consistently deregulated in breast cancer. It is worthy noting that miR-10b, miR-125b, and miR-145, were down-regulated and the other two, miR-21 and miR-155, were up-regulated, which means that they can be treated as tumor suppressor genes or oncogenes, respectively (Iorio et al., 2005). After implementing NRLMFMDA, we can obtained all the rankings for potential miRNA-disease associations from the HMDD v2.0. The final results showed that 8, 16 and 39 of the top 10, 20 and 50 potential miRNAs associated with breast cancer were confirmed, respectively (see Table 2).

**Table 2:** Prediction of the top 50 predicted miRNAs associated with breast neoplasms based on known associations in HMDD database.

**miRNA**	**Evidence**	**miRNA**	**Evidence**
hsa-mir-200c	dbdemc;miR2Disease	hsa-mir-1302	unconfirmed
hsa-let-7e	dbdemc	hsa-let-7i	dbdemc;miR2Disease
hsa-let-7d	dbdemcc;miR2Disease	hsa-mir-133a	dbdemc
hsa-mir-655	unconfirmed	hsa-mir-9	dbdemc;miR2Disease
hsa-mir-590	dbdemc	hsa-mir-103a	unconfirmed
hsa-mir-221	dbdemc;miR2Disease	hsa-mir-450b	unconfirmed
hsa-mir-181	unconfirmed	hsa-mir-19b	dbdemc
hsa-mir-10b	dbdemc;miR2Disease	hsa-mir-18a	dbdemc;miR2Disease
hsa-mir-15a	dbdemc	hsa-mir-23a	dbdemc
hsa-mir-182	dbdemc;miR2Disease	hsa-mir-20b	unconfirmed
hsa-mir-150	dbdemc	hsa-mir-345	dbdemc
hsa-mir-16	dbdemc	hsa-mir-106a	dbdemc
hsa-mir-219	dbdemc	hsa-mir-33a	unconfirmed
hsa-mir-15b	dbdemc	hsa-mir-195	dbdemc;miR2Disease
hsa-mir-17	miR2Disease	hsa-mir-200a	dbdemc;miR2Disease
hsa-mir-422a	dbdemc	hsa-mir-455	dbdemc
hsa-mir-215	dbdemc	hsa-mir-132	dbdemc
hsa-mir-1247	unconfirmed	hsa-mir-652	dbdemc
hsa-mir-151	unconfirmed	hsa-mir-96	dbdemc;miR2Disease
hsa-mir-22	dbdemc;miR2Disease	hsa-mir-1323	unconfirmed
hsa-mir-107	dbdemc	hsa-mir-137	dbdemc
hsa-mir-143	dbdemc;miR2Disease	hsa-mir-202	dbdemc;miR2Disease
hsa-mir-346	dbdemc	hsa-mir-2355	unconfirmed
hsa-mir-191	dbdemc;miR2Disease	hsa-mir-204	dbdemc;miR2Disease
hsa-mir-223	dbdemc	hsa-mir-126	dbdemc;miR2Disease

*The first column records top 1–25 related miRNAs. The second column records the top 26-50 related miRNAs*.

Esophageal Neoplasms is a cancer generated from the esophagus which runs between the throat and the stomach. It is still a common cancer happened among the public. The estimated number of new esophageal cancer cases and deaths were 291238 and 218957, respectively. The crude incidence and mortality rates for esophageal cancer were 21.62/100000 and 16.25/100000, respectively(Zeng et al., 2016a). Researches have showed that low expression of let-7b and let-7c associated with poor response to chemotherapy both clinically and histopathologically, which was observed from 74 patients as the training set in before-treatment biopsies (Sugimura et al., 2012). NRLMFMDA was implemented to identify esophageal neoplasms-associated miRNAs. As a result, 9 out of the top 10 and 40 out of the top 50 predicted esophageal neoplasms related miRNAs were experimentally confirmed by reports (see Table 3).

**Table 3:** Prediction of the top 50 predicted miRNAs associated with esophageal neoplasms based on known associations in HMDD database.

**miRNA**	**Evidence**	**miRNA**	**Evidence**
hsa-mir-146a	dbdemc	hsa-mir-1972	unconfirmed
hsa-mir-26b	dbdemc	hsa-mir-200b	dbdemc
hsa-mir-675	unconfirmed	hsa-mir-20b	dbdemc
hsa-mir-10b	dbdemc	hsa-mir-1247	unconfirmed
hsa-mir-191	dbdemc	hsa-mir-31	dbdemc
hsa-mir-15b	dbdemc	hsa-mir-198	dbdemc
hsa-mir-143	dbdemc	hsa-mir-103a	unconfirmed
hsa-mir-20a	dbdemc	hsa-mir-152	dbdemc
hsa-mir-34b	dbdemc	hsa-mir-1915	unconfirmed
hsa-mir-27a	dbdemc	hsa-mir-195	dbdemc
hsa-mir-9	dbdemc	hsa-mir-320e	unconfirmed
hsa-mir-221	dbdemc	hsa-mir-335	dbdemc
hsa-mir-590	dbdemc	hsa-mir-106b	dbdemc
hsa-mir-200c	dbdemc	hsa-mir-181d	dbdemc
hsa-mir-25	dbdemc	hsa-mir-422a	dbdemc
hsa-let-7a	dbdemc	hsa-mir-372	dbdemc
hsa-mir-203	dbdemc;miR2Disease	hsa-mir-15a	dbdemc
hsa-mir-376c	unconfirmed	hsa-mir-1	dbdemc
hsa-let-7e	dbdemc	hsa-mir-181	unconfirmed
hsa-mir-100	dbdemc	hsa-mir-29a	dbdemc
hsa-mir-29b	dbdemc	hsa-mir-30d	dbdemc
hsa-mir-2355	unconfirmed	hsa-mir-106a	dbdemc
hsa-mir-205	dbdemc;miR2Disease	hsa-mir-92	dbdemc
hsa-mir-30a	dbdemc	hsa-mir-371a	unconfirmed
hsa-mir-125a	dbdemc	hsa-mir-141	dbdemc

*The first column records top 1-25 related miRNAs. The second column records the top 26–50 related miRNAs*.

Lymphoma is a group of blood cell tumors developed from lymphocytes that is a type of white blood cell. It's also worth mentioning that Hodgkin lymphoma and non-Hodgkin lymphoma are the two main types, among which the proportion of patients with non-Hodgkin lymphoma (NHL) is about 90%. (Alizadeh et al., 2000). Experimental studies showed that the miR155 is significantly up-regulated in some Burkitt's lymphoma and several other types of lymphomas (Metzler, 2004). In canine B-cell lymphomas, compared with normal canine peripheral blood mononuclear cells (PBMC) and normal lymph nodes (LN), the expression of miRNA hsa-mir-19a was increased. After the implementation of NRLMFMDA, we took lymphomas as a case study for the identification of potential miRNA-disease association. The results showed that 8 out of top 10 and 37 out of 50 potential lymphoma-associated miRNAs in the prediction result list have been verified based on recent experimental reports (see Table 4).

**Table 4:** Prediction of the top 50 predicted miRNAs associated with lymphoma based on known associations in HMDD database.

**miRNA**	**Evidence**	**miRNA**	**Evidence**
hsa-mir-10b	dbdemc	hsa-mir-106b	dbdemc
hsa-mir-1247	unconfirmed	hsa-let-7a	dbdemc
hsa-mir-221	dbdemc;miR2Disease	hsa-mir-326	dbdemc
hsa-mir-1302	unconfirmed	hsa-mir-99b	dbdemc
hsa-mir-30a	dbdemc	hsa-mir-103a	unconfirmed
hsa-mir-31	dbdemc	hsa-mir-30b	dbdemc
hsa-mir-9	dbdemc	hsa-mir-124	dbdemc
hsa-mir-27b	dbdemc	hsa-mir-204	dbdemc
hsa-mir-181c	dbdemc	hsa-mir-1915	unconfirmed
hsa-let-7d	dbdemc	hsa-mir-410	unconfirmed
hsa-mir-15b	dbdemc	hsa-mir-19b	dbdemc;miR2Disease
hsa-mir-202	unconfirmed	hsa-mir-301b	unconfirmed
hsa-let-7e	dbdemc;miR2Disease	hsa-mir-518a	unconfirmed
hsa-mir-2355	unconfirmed	hsa-mir-125a	dbdemc
hsa-mir-27a	dbdemc	hsa-mir-191	dbdemc
hsa-mir-139	dbdemc;miR2Disease	hsa-mir-23a	dbdemc
hsa-mir-17	dbdemc;miR2Disease	hsa-mir-200c	dbdemc
hsa-mir-215	dbdemc	hsa-mir-33a	dbdemc
hsa-mir-20a	dbdemc;miR2Disease	hsa-mir-1	dbdemc
hsa-mir-29b	dbdemc	hsa-mir-127	dbdemc;miR2Disease
hsa-mir-29c	dbdemc	hsa-mir-132	dbdemc
hsa-mir-208b	unconfirmed	hsa-mir-146b	unconfirmed
hsa-mir-655	unconfirmed	hsa-mir-200b	dbdemc
hsa-mir-99a	dbdemc;miR2Disease	hsa-mir-942	unconfirmed
hsa-mir-219	dbdemc	hsa-let-7b	dbdemc

*The first column records top 1–25 related miRNAs. The second column records the top 26–50 related miRNAs*.

To demonstrate the result of ranking completely, we have provided the prediction list of the whole potential miRNA-disease associations in HMDD v2.0 database and their association scores predicted by NRLMFMDA (see Supplementary Table 1).

In addition, we want to test the prediction ability of NRLMFMDA for the new diseases, namely the ones that have no known association with any miRNA. Therefore, we hid the association information between the miRNAs and the test disease by setting any of the known associations between them as unknown ones. After implementing the NRLMFMDA, we obtained the ranking of the miRNA-disease association prediction scores. We showed the result of hepatocellular carcinoma ranking in Table 5, in which we can see that 9, 18 and 42 related miRNAs out of the top 10, 20, and 50 had been confirmed by at least one of the three databases HMDD, dbDEMC and miR2Disease. Moreover, hsa-mir-146a was ranked first in the top 50 and the recent research has confirmed that a functional polymorphism (rs2910164) in the miR-146a gene is associated with the risk for hepatocellular carcinoma (Xu et al., 2008).

**Table 5:** Prediction of the top 50 predicted miRNAs associated with carcinoma, hepatocellular based on known associations in HMDD database.

**miRNA**	**Evidence**	**miRNA**	**Evidence**
hsa-mir-146a	dbdemc;miR2Disease;HMDD	hsa-mir-1247	unconfirmed
hsa-mir-16	dbdemc;miR2Disease;HMDD	hsa-mir-150	dbdemc;miR2Disease;HMDD
hsa-mir-215	miR2Disease	hsa-mir-483	HMDD
hsa-mir-133b	HMDD	hsa-let-7e	dbdemc;miR2Disease;HMDD
hsa-mir-15a	dbdemc;miR2Disease;HMDD	hsa-mir-205	miR2Disease;HMDD
hsa-mir-15b	dbdemc;HMDD	hsa-mir-139	miR2Disease;HMDD
hsa-mir-103b	unconfirmed	hsa-mir-92a	miR2Disease;HMDD
hsa-mir-345	HMDD	hsa-mir-145	dbdemc;miR2Disease;HMDD
hsa-mir-9	miR2Disease	hsa-mir-204	unconfirmed
hsa-mir-20a	dbdemc;miR2Disease;HMDD	hsa-let-7g	miR2Disease;HMDD
hsa-mir-219	miR2Disease;HMDD	hsa-mir-1302	unconfirmed
hsa-mir-143	dbdemc;miR2Disease	hsa-mir-1972	unconfirmed
hsa-mir-125a	dbdemc;miR2Disease;HMDD	hsa-mir-191	dbdemc;HMDD
hsa-mir-29b	dbdemc;HMDD	hsa-mir-450b	HMDD
hsa-mir-106b	dbdemc;miR2Disease;HMDD	hsa-mir-181d	dbdemc;HMDD
hsa-mir-22	dbdemc;HMDD	hsa-mir-30b	HMDD
hsa-mir-152	miR2Disease;HMDD	hsa-mir-10b	HMDD
hsa-mir-675	unconfirmed	hsa-mir-941	unconfirmed
hsa-mir-27b	dbdemc	hsa-mir-30a	miR2Disease;HMDD
hsa-mir-221	dbdemc;miR2Disease;HMDD	hsa-mir-30d	dbdemc;HMDD
hsa-let-7d	miR2Disease;HMDD	hsa-mir-200a	dbdemc;miR2Disease;HMDD
hsa-mir-100	dbdemc;HMDD	hsa-mir-194	dbdemc;miR2Disease
hsa-mir-26a	dbdemc;miR2Disease;HMDD	hsa-mir-2355	unconfirmed
hsa-mir-198	HMDD	hsa-mir-146b	HMDD
hsa-mir-29a	dbdemc;HMDD	hsa-let-7c	dbdemc;miR2Disease;HMDD

*The first column records top 1–25 related miRNAs. The second column records the top 26–50 related miRNAs*.

Finally, we implemented NRLMFMDA on the old version of the database HMDD to observe whether the model still performs well on it. After implementing the experiment with the proposed method, it had shown the effectiveness on predicting potential miRNA-disease associations based on the previous dataset. For instance, there are 5, 11, and 31 respectively out of top 10, 20, and 50 miRNAs related with the lung neoplasms have been confirmed (see Table 6). As we can see, hsa-mir-96 was ranked first in the top 50 and research has confirmed that the expression of miR-96 in tumors was positively related to its expression in sera. Besides, high expression of tumor and serum miRNAs of the miR-183 family were associated with overall poor survival in patients with lung cancer, which was demonstrated by Log-rank and Cox regression analyses (Zhu et al., 2011).

**Table 6:** Prediction of the top 50 predicted miRNAs associated with lung neoplasms based on known associations in old version HMDD database.

**miRNA**	**Evidence**	**miRNA**	**Evidence**
hsa-mir-96	dbdemc;HMDD	hsa-mir-139	dbdemc;miR2Disease
hsa-mir-498	dbdemc	hsa-mir-323	unconfirmed
hsa-mir-491	unconfirmed	hsa-mir-181d	dbdemc
hsa-mir-335	miR2Disease;HMDD	hsa-mir-379	unconfirmed
hsa-mir-378	unconfirmed	hsa-mir-448	unconfirmed
hsa-mir-596	unconfirmed	hsa-mir-302d	dbdemc
hsa-mir-409	unconfirmed	hsa-mir-301b	unconfirmed
hsa-mir-523	unconfirmed	hsa-mir-1	dbdemc;miR2Disease;HMDD
hsa-mir-526b	dbdemc	hsa-mir-154	dbdemc
hsa-mir-220	miR2Disease	hsa-mir-510	unconfirmed
hsa-mir-15a	dbdemc	hsa-mir-17	miR2Disease;HMDD
hsa-mir-520f	dbdemc	hsa-mir-133a	dbdemc;HMDD
hsa-mir-136	dbdemc;HMDD	hsa-mir-376a	HMDD
hsa-mir-520c	unconfirmed	hsa-mir-219	miR2Disease;HMDD
hsa-mir-657	unconfirmed	hsa-mir-181a	dbdemc;HMDD
hsa-mir-185	dbdemc;HMDD	hsa-mir-25	dbdemc;HMDD
hsa-mir-34a	dbdemc;HMDD	hsa-mir-194	unconfirmed
hsa-mir-514	unconfirmed	hsa-mir-130b	dbdemc
hsa-mir-383	dbdemc	hsa-mir-15b	dbdemc
hsa-mir-642	unconfirmed	hsa-mir-532	unconfirmed
hsa-mir-29a	dbdemc;miR2Disease;HMDD	hsa-mir-598	unconfirmed
hsa-mir-181b	dbdemc;HMDD	hsa-mir-512	unconfirmed
hsa-mir-338	dbdemc;miR2Disease;HMDD	hsa-mir-526a	unconfirmed
hsa-mir-224	dbdemc;miR2Disease;HMDD	hsa-let-7b	miR2Disease;HMDD
hsa-mir-210	dbdemc;miR2Disease;HMDD	hsa-mir-134	HMDD

*The first column records top 1–25 related miRNAs. The second column records the top 26–50 related miRNAs*.

According to the result of case studies on the five major human diseases, excellent prediction performance of NRLMFMDA has been presented. With the development of experimental tools and the improvement of experimental measures, we look forward that more and more miRNA-disease association data verified by experiment will spring up. At that time, increasing portion of the predictions with NRLMFMDA can be verified by researches in the future.

## Discussion

Nowadays, researchers have made progress not only in discovering miRNAs, but also in discovering the important roles that miRNAs play in physiological and pathophysiological processes (Liu and Olson, 2010). For example, aberrant expression of miRNAs has been related with various neurological disorders (NDs) in the central nervous system such as Huntington disease, amyotrophic lateral sclerosis, schizophrenia and autism, Alzheimer disease, Parkinson's disease. If dysregulated miRNAs are discoveried in patients with NDs, this may be used as a biomarker for the earlier diagnosis and monitoring of disease progression (Kamal et al., 2015). MiRNA can also be transcriptional regulators participated in pulmonary sarcoidosis and packaged in extracellular vesicles (EV) during cellular communication (Kishore et al., 2018). In biomedical research, identification of disease-associated miRNAs has become an important filed, which will accelerate people's understanding of disease pathogenesis at the molecular level and disease diagnosis, treatment and prevention in medical(Chen et al., 2017d).

This paper introduced the computational method called NRLMFMDA in which we combined the novel method of logistic matrix factorization with the similarity computational method of Gaussian interaction profile kernel similarity and further assigned higher importance level to the known associations in the process of calculating the potential miRNA-disease association probabilities to assure the larger positive influence of the known data. Additionally, we also took full advantage of the information of nearest neighbor diseases and miRNAs to improve the accuracy of the miRNA-disease association prediction (Liu et al., 2016a). As is known, the logistic matrix factorization technique has been applied in many early work of predicting associations. And it has shown remarkable effectiveness. Taking the neighborhood principle into consideration, we modified it in a more reasonable way to improve the accuracy of prediction. Due to the introduction of the Gaussian interaction profile kernel similarity, the information of the disease similarity and the miRNA similarity was fully excavated to improve the accuracy of the prediction. To verify the accuracy of the NRLMFMDA, three types of cross validation which contains Global LOOCV, Local LOOCV, and 5-fold cross validation have been implemented. As a result, the excellent performance of NRLMFMDA has been showed both from the cross validation and the case studies with several crucial diseases.

Several important factors contribute to the excellent performance of NRLMFMDA. First of all, more and more association pairs between miRNAs and diseases have been discovered and confirmed till now. Due to the data-dependent property of NRLMFMDA, the increasing of known associations assuredly improved the predicting accuracy. Secondly, NRLMFMDA can take full advantage of the similarity information by introducing the Gaussian interaction profile kernel similarity. Thirdly, NRLMFMDA pays attention to the neighborhood information which provides more reliable associations by using the neighborhood regularization method in the training procedure and the neighborhood smoothing method in the final prediction. What's more, some machine learning-based model randomly selected negative samples as training data, this inaccurate chosen process would affect the model's prediction accuracy. The modified latent vectors used in NRLMFMDA can overcome the bias because of using the uncertain negative samples to train the latent vectors of miRNAs and diseases in negative sets, which would helpful to the improvement of prediction accuracy for NRLMFMDA. Last but not least, searching the optimal solution with an alternating gradient ascent procedure made sure the reliability of the disease eigenvectors and the miRNA eigenvectors. In view of above-mentioned, NRLMFMDA has greatly improved the accuracy in prediction association between miRNA and disesase.

Some limitations have been noted in this study. Firstly, though current studies benefit from the increased known data, it is never a finished work to expand data. Numerous excellent methods were proposed just to cover the shortage of the data (Liu et al., 2014; You et al., 2014). Secondly, in the iterative process, we have five parameters that are difficult to choose as the optimal combination. Actually, we have some ranges for the five parameters. However, even using grid search strategy, it wastes a lot of time and resources due to the limitation of current situation. Therefore, we expect to use some optimized search strategy to improve the accuracy of prediction method in the future.

## Author contributions

JQ implemented the experiments, analyzed the result, and wrote the paper. B-SH conceived the project, designed the experiments, analyzed the result, and revised the paper. QZ conceived the project, implemented the experiments, and analyzed the result, and revised the paper. All authors read and approved the final manuscript.

### Conflict of interest statement

The authors declare that the research was conducted in the absence of any commercial or financial relationships that could be construed as a potential conflict of interest.
