# Supplementary material for: Identifying and Exploiting Potential miRNA-Disease Associations With Neighborhood Regularized Logistic Matrix Factorization
Source: Front Genet. 2018 Aug 7;9:303. doi: 10.3389/fgene.2018.00303 (PMC6090164; doi:10.3389/fgene.2018.00303)
Supplement: Supplementary file 2 [file Table_2.DOCX]

**Identifying and exploiting potential miRNA-disease associations with neighborhood regularized logistic matrix factorization**

**Bin-Sheng He^1^, Jia Qu^2, *^, Qi Zhao^3,4,*^**

^1^The First Affiliated Hospital, Changsha Medical University, Changsha, 410219, China

^2^School of Information and Control Engineering, China University of Mining and Technology, Xuzhou, 221116, China

^3^School of Mathematics, Liaoning University, Shenyang, 110036, China

^4^Research Center for Computer Simulating and Information Processing of Bio-Macromolecules of Liaoning Province, Shenyang 110036, China

*** Correspondence:**Jia Qu, Qi Zhao

TB17060015B4@cumt.edu.cn; [zhaoqi@lnu.edu.cn](mailto:zhaoqi@lnu.edu.cn)

**Keywords: microRNA, disease, association prediction, matrix factorization**

**1 Supplementary Table**

**Supplementary Table 1.** We applied NRLMFMDA to prioritize all the candidate miRNA-disease pairs based on all the known miRNA-disease associations in HMDD database as training samples. This prediction result is released for further experimental validation and research.
